# O Gene da Paraoxonase 1 (PON1) no Contexto Doença Arterial Coronariana

**DOI:** 10.36660/abc.20220645

**Published:** 2022-10-05

**Authors:** Denise da Silva Pinheiro, Rosália Santos Amorim Jesuíno

**Affiliations:** 1 Universidade Federal de Goiás Instituto de Ciências Biológicas Goiânia GO Brasil Universidade Federal de Goiás - Instituto de Ciências Biológicas , Goiânia , GO – Brasil

**Keywords:** Doença Arterial Coronariana/genética, Biomarcadores, Predisposição Genética para Doença, Estudos de Associação Genética, Aridialquilfosfatase/genética, Lipoproteínas/sangue, Paraoxonase 1 (PON1), Polimorfismo de Nucleotídeo Único

As doenças cardiovasculares (DCV), e mais especificamente a doença arterial coronariana (DAC), continuam como as mais importantes causas de mortes por doenças não-transmissíveis no Brasil e no mundo. ^1 , 2^ De forma que esforços científicos têm sido empreendidos na identificação de novos marcadores bioquímicos e na elucidação de perfis genotípicos de risco a DAC e outras DCV no campo da genética médica de associação a doenças. ^3^ Nesse sentido, um gene que tem sido extensivamente estudado é o da paraoxonase (PON) , o qual apresenta três isoformas em cluster de genes: PON1, PON2 e PON3, com localização no cromossomo 7q21.3-22.1. ^4^ PON1 constitui o membro da família de paraoxonases mais estudado devido ao seu destacado papel em vias de catabolismo de lipoproteínas, sendo inclusive apontada como um marcador bioquímico da capacidade antioxidante das partículas de HDL-colesterol. ^5 , 6^

PON1 é uma enzima éster hidrolase cálcio-dependente multifuncional que se encontra associada às partículas de HDL-colesterol. Apresenta propriedades antioxidantes e antiaterogênicas por hidrolisar o colesterol da LDL oxidado e produtos da peroxidação de fosfolipídios. Desta forma, confere proteção às membranas celulares e neutraliza os efeitos da oxidação lipídica, desempenhando importante papel cardioprotetivo. ^4 , 7^

Polimorfismos das enzimas paraoxonases, particularmente a isoforma PON1, têm sido associadas com alterações lipídicas ^8^ e implicadas na patogenia da DAC conforme demonstrado em alguns estudos, ^9 , 10^ embora deva-se ressaltar a heterogeneidade de resultados na literatura conforme apontado em amplos estudos de metanálise, que concluíram pela associação fraca ou ausente para os principais polimorfismos estudados. ^11 , 12^ Dentre os quais, ressalta-se dois polimorfismos na região codificante do gene, com substituição na proteína de Glutamina (Q) por Arginina (R) na posição 192 (rs662 ou A192G) e Leucina por Metionina na posição 55 (rs854560 ou A55T) e o polimorfismo rs705379 (ou T[-107]C) na região promotora do gene, os quais tem sido relatados por influenciar a atividade ou a expressão da enzima.

Neste contexto, deve-se destacar o trabalho de Soflaei et al., ^13^ que avaliaram a associação dos polimorfismos da PON1 mencionados (rs662, rs854560, rs705379) com DAC na população do Irã da região nordeste do país, pela comparação entre pacientes com DAC definida angiograficamente (grupo com angiografia positiva - obstrução com estenose coronariana >50% em pelo menos uma artéria coronária [N=266] e grupo com angiografia negativa - não obstrução com estenose < 30% nas artérias coronárias [N=335]). Os resultados obtidos indicaram uma significativa associação do alelo G (isoforma 192R da PON1) do polimorfismo rs662 com o aumento do risco a doença (genótipo GG: OR = 2,424, IC 95% [1,123-5,233]; Alelo G: OR = 1,663, IC 95% [1,086-2,547]), apresentando consistência com resultados de outros estudos, ^9 , 10^ inclusive com outro estudo conduzido no oeste do Irã, ^14^ e com achados de metanálise. ^11 , 12^

O referido estudo também explorou a associação da atividade da PON1 (fenótipo) com DAC, não tendo verificado diferença entre os grupos estudados, porém confirmou o efeito dos polimorfismos avaliados sobre o nível de atividade dosada da PON1, tendo observado maior atividade da paraoxonases, que seria benéfico, nos portadores do perfil genotípico de risco (alelo G ou genótipo GG de rs662). Este achado aparentemente paradoxal de avaliação de genótipo de risco e perfil de atividade da PON1 têm sido explicado, conforme discutido no estudo, pela diferença entre o que é medido como atividade da enzima no ensaio bioquímico de hidrólise do paraoxon (atividade paraoxonase) e sua atividade de proteção antioxidante em relação ao LDL-colesterol, que envolve outro sítio ativo na enzima, de forma que os portadores do alelo G (isoforma 192R) teriam, na verdade, uma atividade biológica reduzida da PON1 no que se refere a proteção antioxidante, como evidenciado no estudo de Aviram et al., ^15^ sinalizando um aspecto importante a ser considerado nos estudos de atividade desta enzima, devido ao seu caráter multifuncional.

De forma relevante na realidade atual, os estudos de associação genética têm permitido o desenvolvimento da medicina personalizada pela aplicação dos achados científicos obtidos na construção de painéis de testes genéticos por grupos de doenças, incluindo painéis específicos para as DCV, que já se encontram inclusive disponíveis à população em alguns laboratórios especializados. Os avanços no nível de conhecimento relacionados ao efeito de variantes genéticas em mecanismos moleculares subjacentes à fisiopatologia de doenças, como tem sido observado para os genes da paraoxonase na DAC, têm o potencial de propiciar o desenvolvimento de painéis genéticos mais acurados de forma a impactar decisões terapêuticas e abordagens no aconselhamento genético.

## References

[1] Oliveira GM, Brant LC, Polanczyk A, Carisi A, Biolo A, Nascimento BR (2020). Cardiovascular statistics–brazil 2020. Arq Bras Cardiol.

[2] World Health Organization (WHO) Global Health Estimates 2020: Deaths by Cause, Age, Sex, by Country and by Region.

[3] Izar MC, Helfenstein Fonseca FA, Miki Ihara SS, Kasinski N, Won Han S, Lopes IE (2003). Risk Factors, biochemical markers, and genetic polymorphisms in early coronary artery disease. Arq Bras Cardiol.

[4] Primo-Parmo SL, Sorenson RC, Teiber J, la Du BN (1996). The Human Serum Paraoxonase/Arylesterase Gene (PON1) Is One Member of a Multigene Family. Genomics.

[5] Garin MCB, Moren X, James RW (2006). Paraoxonase-1 and serum concentrations of HDL-cholesterol and apoA-I. J Lipid Res.

[6] Dullaart RPF, Otvos JD, James RW (2014). Serum paraoxonase-1 activity is more closely related to HDL particle concentration and large HDL particles than to HDL cholesterol in Type 2 diabetic and non-diabetic subjects. Clin Biochem.

[7] Mackness MI, Mackness B, Durrington PN, Fogelman AM, Berliner J, Lusis AJ (1998). Paraoxonase and coronary heart disease. Curr Opin Lipidol.

[8] Scherrer DZ, Zago VHS, Vieira IC, Parra ES, Panzoldo N, Alexandre F (2015). O SNP p.Q192R da PON1 não Parece Associar-se a Fatores de Risco para a Aterosclerose Carotídea em Amostra de População Brasileira Normolipidêmica e Assintomática. Arq Bras Cardiol.

[9] Munshi R, Panchal F, Chaurasia A, Rajadhyaksha G (2018). Association between Paraoxonase 1(PON1) Gene Polymorphisms and PON1 Enzyme Activity in Indian Patients with Coronary Artery Disease (CAD). Curr Pharmacogenomics Person Med.

[10] Liu T, Zhang X, Zhang J, Liang Z, Cai W, Huang M (2014). Association between PON1 rs662 polymorphism and coronary artery disease. Eur J Clin Nutr.

[11] Wang M, Lang X, Zou L, Huang S, Xu Z (2011). Four genetic polymorphisms of paraoxonase gene and risk of coronary heart disease: A meta-analysis based on 88 case–control studies. Atherosclerosis.

[12] Ashiq S, Ashiq K (2021). The Role of Paraoxonase 1 (PON1) Gene Polymorphisms in Coronary Artery Disease: A Systematic Review and Meta-Analysis. Biochem Genet.

[13] Soflaei SS, Baktashian M, Moghaddam KH, Saberi-Karimian M, Koseri N, Hashemi SM (2022). Association of Paraoxonase-1 Genotype and Phenotype with Angiogram Positive Coronary Artery Disease. Arq Bras Cardiol.

[14] Vaisi-Raygani A, Ghaneialvar H, Rahimi Z, Tavilani H, Rourmotabbed T, Shakiba E (2011). Paraoxonase Arg 192 allele is an independent risk factor for three-vessel stenosis of coronary artery disease. Mol Biol Rep.

[15] Aviram M, Billecke S, Sorenson R, Bisgaier C, Newton R, Rosenblat M (1998). Paraoxonase Active Site Required for Protection Against LDL Oxidation Involves Its Free Sulfhydryl Group and Is Different From That Required for Its Arylesterase/Paraoxonase Activities. Arterioscler Thromb Vasc Biol.

