# Supplementary material for: O Gene da Paraoxonase 1 (PON1) no Contexto Doença Arterial Coronariana
Source: Arq Bras Cardiol. 2022 Oct 5;119(4):602–3. [Article in Portuguese] doi: 10.36660/abc.20220645 (PMC9563891; doi:10.36660/abc.20220645)
Supplement: Translation

# Translation: The Paraoxanase 1 (PON1) Gene in the Context of Coronary Artery Disease

**Authors:** Denise da Silva Pinheiro, Rosália Santos Amorim Jesuíno

**Affiliations:** 1 Brazil Universidade Federal de Goiás - Instituto de Ciências Biológicas,Goiânia, GO – Brazil

**Keywords:** Coronary Artery Disease/genética, Biomarkers, Genetic Predisposition to Disease, Genetic Association Studies, Aryldialkylphosphatase/genetics, Lipoproteins/blood, Paraoxanase 1 (PON 1), Polymorphism, Single Nucleotide

Cardiovascular Diseases (CVD), and more specifically Coronary Artery Disease (CAD), continue to be the most important causes of death from non-communicable diseases in Brazil and in the world.^1 , 2^ Therefore, scientific efforts have been made to identify new biochemical markers and in the elucidation of genotypic risk profiles for CAD and other CVDs in the field of disease-association medical genetics.^3^ In this sense, a gene that has been extensively studied is the paraoxanase (PON) gene, which has three gene cluster isoforms: PON1, PON2 and PON3, located on chromosome 7q21.3-22.1.^4^ PON1 is the most studied member of the paraoxanases family due to its prominent role in lipoprotein catabolism pathways, being even pointed out as a biochemical marker of the antioxidant capacity of HDL-cholesterol particles.^5 , 6^

PON1 is a multifunctional calcium-dependent ester hydrolase enzyme that is associated with HDL-cholesterol particles. It has antioxidant and antiatherogenic properties by hydrolyzing oxidized LDL cholesterol and phospholipid peroxidation products. In this way, it provides protection to cell membranes and neutralizes the effects of lipid oxidation, playing an important cardioprotective role.^4 , 7^

Polymorphisms of paraoxonase enzymes, particularly the PON1 isoform, have been associated with lipid alterations^8^ and have been implicated in the pathogenesis of CAD, as demonstrated in some studies,^9 , 10^ although the heterogeneity of results in the literature should be highlighted, as pointed out in extensive meta-analysis studies, who concluded that there was a weak or absent association for the main polymorphisms studied.^11 , 12^ Among which, two polymorphisms in the coding region of the gene stand out, with replacement of the Glutamine (Q) by Arginine (R) at protein position 192 (rs662 or A192G) and Leucine by Methionine at position 55 (rs854560 or A55T) and rs705379 polymorphism (or T[-107]C) in the promoter region of the gene, which have been reported to influence enzyme activity or expression.

In this context, it is worth highlighting the work of Soflaei et al.,^13^ who evaluated the association of the mentioned PON1 polymorphisms (rs662, rs854560, rs705379) with CAD in the population of Iran in the northeast region of the country, by comparing patients with angiographically defined CAD (group with positive angiography - obstruction with stenosis coronary artery disease >50% in at least one coronary artery [N=266] and group with negative angiography - non-obstruction with stenosis < 30% in coronary arteries [N=335]). The results obtained indicated a significant association of the G allele (192R isoform of PON1) of the rs662 polymorphism with increased disease risk (GG genotype: OR = 2.424, 95% CI [1.123-5.233]; G allele: OR = 1.663, 95% CI [1.086-2.547]), which is consistent with results from other studies,^9 , 10^ including another study conducted in western Iran,^14^ and with meta-analysis findings.^11 , 12^

The aforementioned study also explored the association of PON1 activity (phenotype) with CAD, not finding any difference between the groups studied, but it confirmed the effect of the polymorphisms evaluated on the level of measured activity of PON1, having observed greater activity of paraoxanase, which would be beneficial, in carriers of the genotypic risk profile (G allele or GG genotype of rs662). This apparently paradoxical finding of risk genotype assessment and PON1 activity profile has been explained, as discussed in the study, by the difference between what is measured as enzyme activity in the biochemical paraoxon hydrolysis assay (paraoxanase activity) and its activity of antioxidant protection in relation to LDL-cholesterol, which involves another active site in the enzyme, so that carriers of the G allele (192R isoform) would actually have a reduced biological activity of PON1 with regard to antioxidant protection, such as evidenced in the study by Aviram et al.,^15^ indicating an important aspect to be considered in studies of the activity of this enzyme, due to its multifunctional character.

Currently, genetic association studies have allowed the development of personalized medicine through the application of scientific findings obtained in the construction of panels of genetic tests by groups of diseases, including specific panels for CVD, which are already available to the public in some specialized laboratories. Advances in the level of knowledge related to the effect of genetic variants on molecular mechanisms underlying the pathophysiology of diseases, as has been observed for the paraoxanase genes in CAD, have the potential to foster the development of more accurate genetic panels to impact therapeutic decisions and approaches in genetic counseling.
